# Reduced plasma levels of soluble interleukin-7 receptor during graft-versus-host disease (GVHD) in children and adults

**DOI:** 10.1186/1471-2172-15-25

**Published:** 2014-06-19

**Authors:** Thomas Poiret, Lalit Rane, Mats Remberger, Birgitta Omazic, Åsa Gustafsson-Jernberg, Nalini Kumar Vudattu, Raija Ahmed, Ingemar Ernberg, Jacek Winiarski, Isabelle Magalhaes, Olle Ringden, Markus Maeurer

**Affiliations:** 1 Division of Therapeutic Immunology, Labmed, Karolinska Institutet, Stockholm, Sweden; 2 Department of Microbiology, Tumor and Cell Biology, Karolinska Institutet, Stockholm, Sweden; 3 Center for allogeneic stem cell transplantation (CAST), Karolinska University Hospital, Stockholm, Sweden; 4 Department of Pediatrics, Astrid Lindgen Barnsjukhuset, Karolinska Hospital Stockholm, Stockholm, Sweden; 5 Department of Immunobiology and Internal Medicine, Yale University, New Haven, USA; 6 The Public Health Agency of Sweden, Sweden; 7 Therapeutic Immunology, F79, LabMed, Hälsovägen, Karolinska University Hospital Huddinge, SE-14186 Huddinge, Sweden

**Keywords:** HSCT, Interleukin-7, Interleukin-7 receptor, Immune reconstitution, CMV, GVHD

## Abstract

**Background:**

Interleukin 7 (IL-7) signals via the IL-7 receptor (IL-7R) and drives homeostatic T-cell proliferation in patients after allogeneic hematopoietic stem cell transplantation (aHSCT).

**Purpose:**

We performed a prospective study in adults (n = 33) and children (n = 29) undergoing aHSCT measuring plasma IL-7 and soluble IL-7R (sIL-7R) concentrations between 1 and 12 months after HSCT in order to investigate the link between sIL-7R and clinical events after aHSCT.

**Results:**

sIL-7R, but not IL-7, increased with time after HSCT in plasma from all patients enrolled in the study. sIL-7R values were higher at 2, 3, and 6 months (p < 0.01) if the donor was a sibling as compared to an unrelated donor. Increased sIL-7R levels were also identified in plasma from patients who were not treated with anti-thymocyte globulin (ATG). Low sIL-7R was associated with any grade of acute graft-versus-host disease (GVHD) at 2 and 6 months (p = 0.02) and with a positive CMV PCR at 2 months after HSCT (p < 0.05). Patients with cytomegalovirus (CMV) reactivation had increased IL-7 values at 2 and 3 months (p = 0.02) after HSCT. In multivariate analysis, lower sIL-7R levels were associated with acute GVHD (relative hazard (RH): 0.70, p > 0.01) and sibling donors (RH: 2.23, p = 0.004). Recipients of sibling grafts showed high levels of IL-7 (RH: 1.38, p < 0.05) and bone marrow recipients had low IL-7 levels (RH: 0.73, p = 0.04).

**Conclusions:**

Measurement of the sIL-7R/IL-7 axis will help in guided immune monitoring after HSCT and guided interference with sIL-7R may be explored in GVHD management.

## Background

Interleukin-7 (IL-7) is a key cytokine in allogeneic stem cell transplantation (aHSCT) and drives homeostatic, extrathymic T-cell expansion in lymphopenic hosts [1]. IL-7 may also promote expansion of alloreactive T-cells that mediate graft-versus-host disease (GVHD) [2]. Elevated IL-7 levels in serum have been shown to be associated with acute GVHD [3,4]. Several cell types have been described to contribute to IL-7 production, e.g. stromal cells [5], macrophages [6], B-cells [7] and thymic epithelial cells. Several conditions may impair IL-7 production, including infections associated with tissue damage of stromal cells, the fibroblastic reticular network (FRC) [8] and cytotoxic therapies in the course of anti-cancer treatment or in conditioning regimens for HSCT. Murine studies have shown that radiation, provided in the course of HSCT, can result in reduced thymic stromal IL-7 production [9]. However, it has not been unequivocally proven that thymus-derived IL-7 contributes substantially to systemic IL-7 levels. IL-7 is consumed by the pool of available immune cells expressing the heterodimeric IL-7 receptor (IL-7R, CD127) along with the common gamma chain (CD132) [1,3]. Expression of the IL-7R on T-cells is associated with T-cell differentiation and maturation, based on CD45RA and CCR7 expression [10]. Precursor T-cells exhibited the highest numbers of IL-7R molecules per cell and terminally differentiated T-cells were found to express the lowest number of IL-7R molecules per cell.

The role of IL-7 in immune reconstitution after HSCT is multifaceted: it promotes thymopoiesis by driving development of immature thymocytes [11,12]. Some reports have suggested that IL-7 treatment leads to improved—but transient—immune reconstitution [11] without increased alloreactivity [13,14]. In contrast, other studies have shown that IL-7 aggravates GVHD [2] and that subsequent blockade of the alpha chain of the IL-7R may prevent GVHD [15]. The situation is even more complex, since the IL-7R is not only available in the cell-membrane-bound format, but also as a soluble form (sIL-7R). The soluble IL-7R binds IL-7 with an affinity similar to that of the membrane bound IL-7R [16], leading to sIL-7R-mediated inhibition of IL-7 signaling in T-cells [17]. sIL-7R is not only generated by shedding of membrane-bound receptors, it is also associated with polymorphism in the IL-7R gene (rs6897932), which leads to increased splicing in the transmembrane domain of exon 6 in the 8-exon Il-7R gene [18-20] resulting in increased sIL-7R generation. This SNP has been associated with autoimmune diseases, i.e. type-I diabetes mellitus [21] and rheumatoid arthritis [22]. IL-7R polymorphism has also been studied in adults after HSCT with inconclusive results concerning SNP analysis of donors [23] and recipients [24]. Up to now, the protein concentrations of IL-7 combined with its receptor IL-7R have not been measured in plasma from children and adults during 12 months after HSCT. We therefore designed a longitudinal study to determine IL-7/IL-7R plasma levels in 61 individuals after HSCT and we investigated associations between IL-7/IL-7R and clinical events after HSCT.

## Methods

### Patients and controls

The study involved 29 children and 32 adults (Table 1). Forty-eight patients underwent HSCT for malignant disorders, 16 patients were in first complete remission. 21 patients received grafts from HLA-identical sibling donors and the remaining patients received grafts from matched unrelated donors (n = 33), or HLA-mismatched unrelated donors (n = 8). The sources of stem cells were bone marrow, peripheral blood stem cells, or in a few individuals, cord blood transplants. Genomic HLA typing (MHC class I and class II four-digit typing) was performed as described previously [25]. IRB approval (Stockholm Ethical Committee South 2010/760-31/1) was in place. Peripheral blood mononuclear cells (PBMCs) were from adult participants (n = 32) and in the case of children (n = 29), consent was obtained from their parents or legal guardians (on file at CAST; Center for allogeneic stem cell transplantation). Samples were obtained from 61 patients at 1, 2, 3, 6, and 12 months after HSCT and 26 controls (15 adults and 11 children). Plasma was obtained after centrifugation and stored at -20°C; PBMCs were isolated from heparinized blood over a ficoll hypaque gradient. The cells were preserved in liquid nitrogen using fetal bovine serum (FBS) containing 10% DMSO.

**Table 1:** Summary of patient characteristics

**Characteristics**	**N = 61**
**Age**	18 (<1–65)*
**Children (<18 y)**	29
**Sex (M/F)**	38/23
**Diagnosis:**	
Non-malignant	13
Acute myeloid leukemia/Acute lymphoid leukemia	12/13
Chronic lymphoid leukemia	4
Myelodysplastic syndrome	11
Other malignancies	8
**Stage (early/late)**	16/32
**Donor age**	26 (0–62)
**Donor sex (M/F)**	38/23
**Donor**	
Sibling/HLA-identical, related	21
MUD	32
HLA-mismatched, unrelated	8
**Conditioning**	
MAC/RIC	34/27
Chemo-based	38
TBI-based	23
**ATG**	47 (77%)
**GVHD prophylaxis**	
CsA ± MTX	41
CsA + Prednisolon	3
Tacrolimus + Sirolimus	16
CsA + MTX + Cy	1
**Stem cell source:**	
BM/PBSCs/CB	25/31/5

*Median [4].

*Abbreviations*: *MUD* matched unrelated donor, *MAC* myeloablative conditioning, *RIC *reduced-intensity conditioning, *TBI* total body irradiation, *ATG* anti-thymocyte globulin, *GVHD* graft-versus-host disease, *CsA* cyclosporine, *MTX* methotrexate, *Cy* cyclophosphamide, *BM* bone marrow, *PBSCs* peripheral blood stem cells, *CB* cord blood.

### HSCT regimen

#### Conditioning

Conventional myeloablative conditioning was given to 34 patients and consisted of cyclophosphamide (Cy) at 60 mg/kg for two days in combination with fractionated TBI (FTBI) at 3 Gy/day for four days (n = 15), or busulphan (Bu) at 4 mg/kg/day for four days (n = 17) [26]. Two patients received other protocols. Reduced-intensity conditioning (RIC) was given to 27 patients and consisted of fludarabine (Flu) at 30 mg/m^2^ for 3–6 days in combination with either Bu at 4 mg/kg/day for two days (n = 7), FTBI at 3 Gy/day for two days and Cy at 60 mg/kg/day for two days (n = 7), Cy at 30 mg/kg/day for two days (n = 7), treosulphan at 12–14 g/m^2^/day for 3 days (n = 5), or TBI (2 Gy) (n = 1).

#### GVHD prophylaxis and CMV PCR

Immunosuppressive treatment mainly consisted of cyclosporine (CsA) in combination with a short course of methotrexate (MTX) (n = 41), or tacrolimus and sirolimus (n = 16) [27]. All patients with an unrelated donor or a non-malignant disease received anti-thymocyte globulin (ATG) (Thymoglobulin, Genzyme, Cambridge, MA) (n = 45) or alemzumab (Genzyme) (n = 2) for 2–4 days during conditioning [28]. During the first month, blood CsA levels were kept at 100 ng/mL in patients with malignancies when a sibling donor was used and at 200–300 ng/mL when an unrelated donor was used and also in patients with non-malignant disorders regardless of the donor. In the absence of GVHD, CsA was discontinued after three to six months for patients with malignancies and after 12–24 months for patients with non-malignant disorders. Patients were monitored for CMV viral load with a quantitative PCR on whole blood from the time of engraftment weekly until day 100 after HSCT as published previously. Later than three months after HSCT, weekly monitoring was continued only in those patients who had experienced CMV reactivation or had severe GVHD, while the other patients were monitored at each visit to the transplant center occurring every 2-3 weeks until 6 months after HSCT. Pre-emptive antiviral treatment with either i.v. ganciclovir 5 mg/kg BID or oral valganciclovir 900 mg BID was given at the center’s chosen intervention limit > 1000 copies/mL blood. The duration of therapy was a minimum of two weeks and was discontinued when the CMV viral load was < 500 copies/mL [29,30].

#### Supportive care

All patients were kept in reversed isolation or they were treated at home, as described in detail previously [31].

### Statistical analysis

Differences between patient groups (i.e. children versus adults) or within each group (i.e. comparing different time points) were analyzed using Mann-Whitney U-test or the Wilcoxon test using the Statistical software program (version 10) and GraphPad Prism 4 software. In the multivariate analysis of factors affecting the levels of sIL-7R at different time points, multiple regression were used. To determine whether there was any correlation between CD127 (IL-7R) positive immune cells and soluble IL-7R levels, we used linear regression analysis with the GraphPad software.

### Quantification of plasma IL-7 and IL-7R

IL-7 quantification was performed using the ELISA IL-7 Eli-pair (Cat. 851.680.010; Cell Sciences, Inc., Canton, MA) according to the manufacturer’s protocol (standard range between 200 and 3,125 pg/mL). Soluble CD127 was measured using an IL-7R ELISA; the anti-IL-7R alpha chain-directed antibody R34.34 (anti-CD127 purified Ab, 1 μg/ml; Beckman Coulter Inc., Brea CA) served as the capture antibody (50 μL/well) by incubation overnight with plasma at 4°C. The recombinant human IL-7 R alpha/CD127 Fc Chimera (306-IR; R&D Systems, Minneapolis, MN) served as the standard (ranging between 0.78125 and 100 ng/mL). Standard and samples were incubated for 4 h, followed by washing steps (PBS, 0.05% Tween) as described earlier [16]. sIL-7R was detected with a biotinylated anti-CD127 antibody (BAF306; R&D Systems). Incubation was for 1 h at RT, followed by washing as described above. Streptavidin-HRP was applied (554066: BD Biosciences, Frankin Lakes, NJ) for 30 min, with subsequent development using Tetramethylbenzidine (TMB). The absorbance was read at 450 nm.

### Flow cytometry

PBMCs (0.5 × 10^6^ cells) were first stained with anti-CCR7 for 15 min at 4°C, followed by addition of the 10-color antibody mix as described in detail previously [10]. The PBMC-antibody mixture was incubated for 15 min at 4°C. The anti-CD27 antibody was then added to the cells, which were incubated at 4°C for 15 min, followed by washing with 1 mL of PBS containing 0.1% BSA. The cell pellet was resuspended in 200 μL of PBS (with 0.1% BSA) and the cells were analyzed as described previously [10].

For analysis of PBMCs from children, frozen PBMCs were thawed and 1 × 10^6^ cells were incubated at 4°C for 15 min with the following antibodies: peridinin-chlorophyll-protein complex- (PerCP-) conjugated anti-CD3 (SK7), allophycocyanine 7- (APC-Cy7-) conjugated anti-CD8α chain (SK1), phycoerythrincyanin 7- (PE-Cy7-) conjugated anti-CCR7 (3D12) purchased from BD Biosciences (Stockholm, Sweden), Krome Orange-conjugated anti-CD4 (13B8.2), fluorescein isothiocyanate- (FiTC-) conjugated anti-CD8β chain (2ST8.5H7), APC-Alexa Fluor 700-conjugated anti-CD107a (H4A3), PE-Texas Red-conjugated anti-CD45RA (2H4), APC-conjugated anti-CD127 (R34.34) purchased from Beckman Coulter (Marseille, France), and Brilliant Violet-conjugated anti-CD117 (104D2) purchased from BioLegend (London, UK). After washing, flow cytometric analysis was performed using a Navios flow cytometer (Beckman Coulter, Miami, FL, USA) and data were analyzed by using FlowJo software (Tree Star Inc., Ashland, OR; USA).

## Results

### Different dynamics of immune reconstitution in children and adults after HSCT

**Figure 1:**
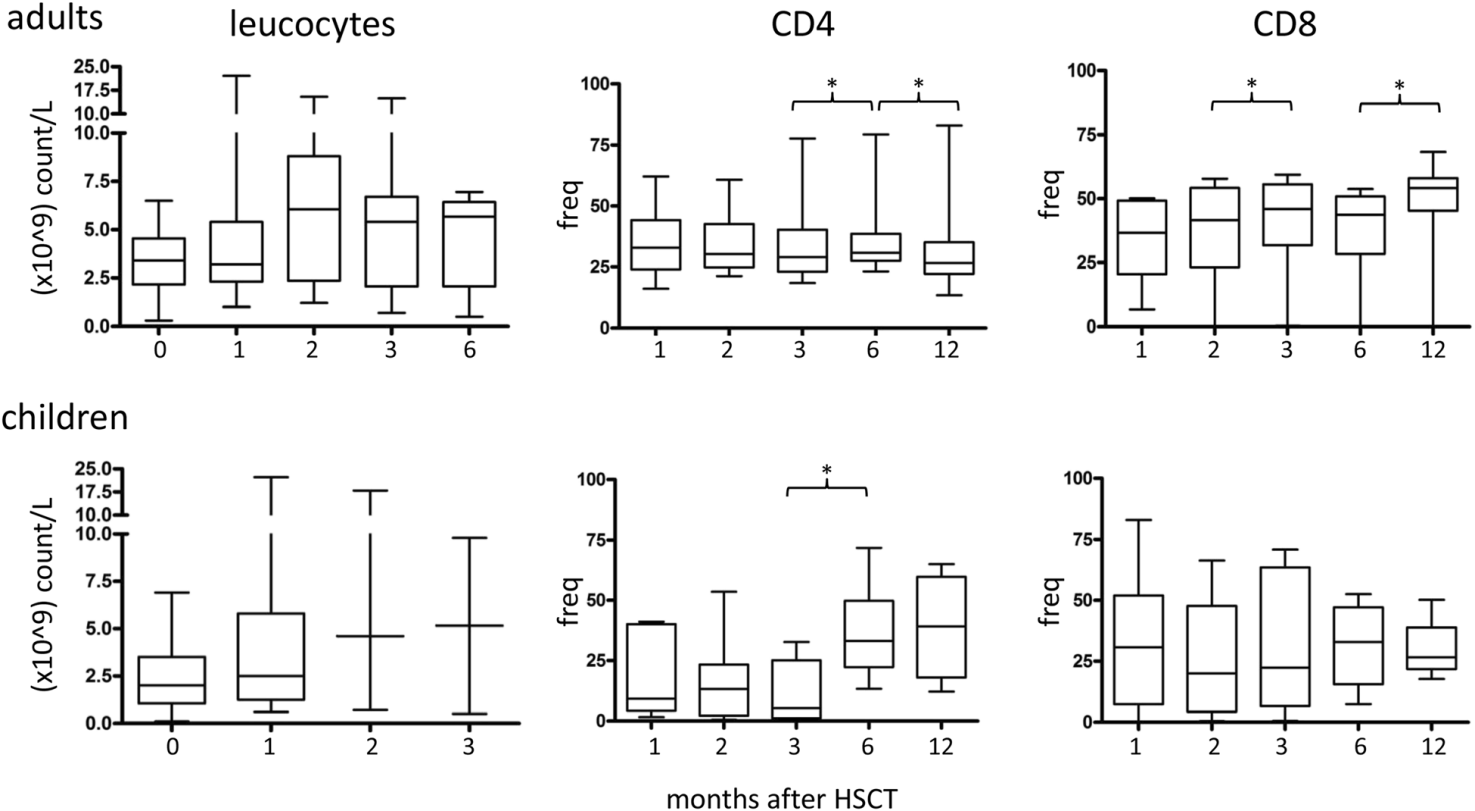
**Immune reconstitution measured as absolute leukocyte counts (left figures) and CD4 and CD8 T-cell frequency after HSCT in blood from adults and children.** * = p < 0.05. Note that leukocyte counts were available from children at the time of transplantation (0) and 1–3 months after HSCT.

sIL-7R and IL-7 were determined at different time points after HSCT in biological material from thirty-two adults and twenty-nine children (Table 1). The frequency of CD4^+^ cells (in CD3^+^ T-cells) between 1 and 12 months after HSCT was 30% for adults after aHSCT (Figure 1), the frequency of CD3^+^CD4^+^ T-cells was lower in children in months 1–3 (first month, p = 0.01; second month, p = 0.003; and third month, p = 0.01) after HSCT as compared to adult HSCT recipients. PBMCs from children also showed a not-significant trend of a lower frequency of CD3^+^CD8^+^ T-cells in PBMCs, compared to PBMCs from adults, at months 1–3 after HSCT (Figure 1, bottom panel) with a proportionate increase in CD3^+^CD4^-^CD8^-^ T-cells in PBMCs (data not shown). PBMCs from children exhibited lower leukocyte counts than adults prior to transplantation (p = 0.02). There were no significant differences in proportion of CD8^+^ cells between PBMCs from children and before and after HSCT.

**Figure 2:**
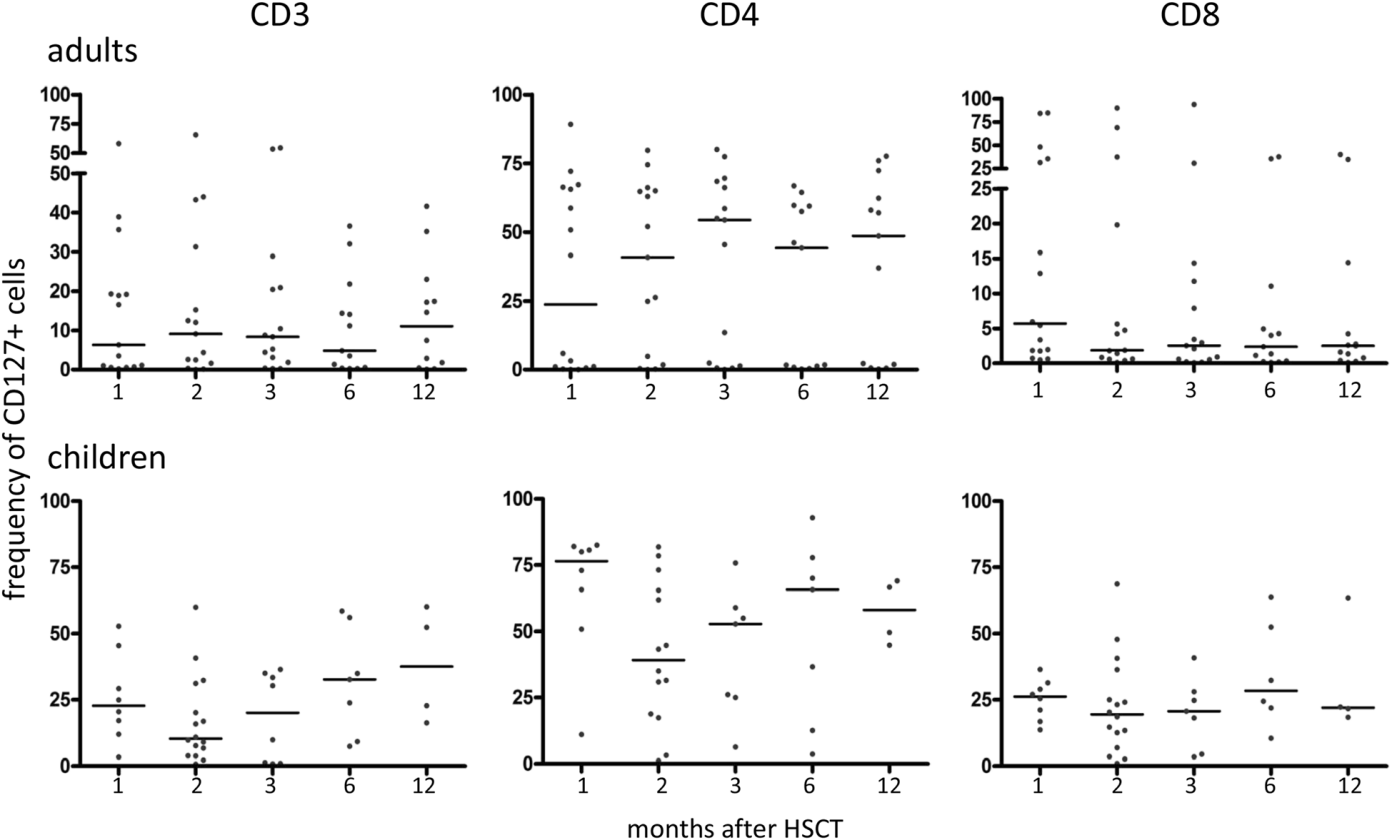
**Percentage of CD127**^**+ **^**(IL-7R**^**+**^**) T-cells in patients after HSCT.** Blood was taken at different time points after HSCT and CD127 expression was analyzed by flow cytometry on CD3^+^, CD3^+^CD4^+^, and CD3^+^CD8^+^ T-cells in PBMCs from adults and children.

PBMCs from children showed a higher frequency of the CD127^+^ (IL-7R^+^) subpopulation in CD4^+^ and CD8^+^ T-cells as compared to adults (Figure 2). The frequency of CD127^+^CD3^+^ cells (total CD3^+^ T-cells) was lower at 6 months (p = 0.02) in adults as compared to children and the median for the CD127^+^CD4^+^ T-cell population at 1 month after HSCT was 76.5% as compared to 23.7% in adults (p < 0.05). A different situation was found to be true for the frequency of the CD127^+^CD8^+^ population, which was lower in PBMCs from adults at 2 months (p = 0.03), 3 (p = 0.02), 6 (p = 0.01), and at 12 months (p = 0.03) as compared to children.

### Dynamics of sIL-7R and IL-7 in adults and children after HSCT

**Figure 3:**
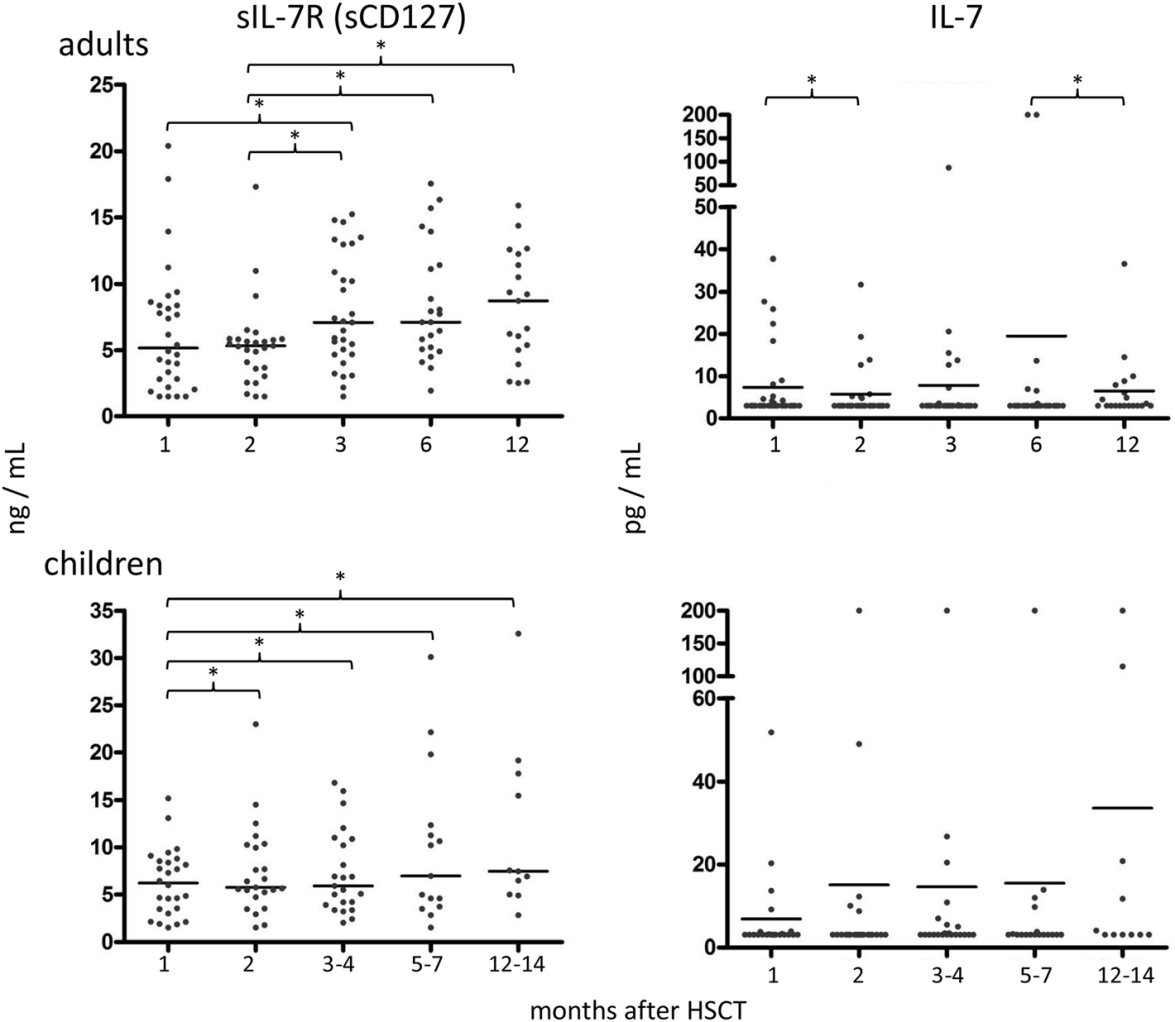
**Levels of soluble Il-7R (sCD127, left figures) and IL-7 (right figures) in plasma from adults and children after HSCT.** * = p < 0.05. Plasma was obtained at different time points after HSCT and examined for IL-7 and sIL-7R by ELISA. Median levels are marked with a bar. sIL-7R levels increased with time after HSCT (non-parametric Wilcoxon test). Differences in plasma from children: 2 months after aHSCT as compared to 1 month (p = 0.02), 3–4 months vs. 1 month (p = 0.04), 5–7 months vs. 1 month (p = 0.02) and 12–14 months vs. 1 month (p = 0.03).

The median sIL-7R protein content in plasma from adults was 5.17 ng/mL at 1 month after HSCT, it increased to 8.72 ng/mL at 12 months. In plasma from children, the median sIL-7R protein concentration was 5.43 ng/mL at 1 month after HSCT and 7.46 ng/mL at 12 months (Figure 3). No significant differences in plasma levels of sIL-7R or IL-7 were found in samples from adults and children. Significant differences concerning the sIL-7R in plasma from adults were seen at 3 months relative to 1 month (p = 0.03), 3 months relative to 2 months (p = 0.04), 6 months relative to 2 months (p = 0.002) and 12 months relative to 2 months (p = 0.03). In plasma from children, the sIL-7R protein levels increased at 5–7 months (p = 0.02) and at 12–14 months (p = 0.03), relative to the first month after HSCT. The median sIL-7R values in plasma from controls (adults and children) were respectively 9.55 ng/mL and 95.05 ng/mL (see online Additional file 1: Figure S1, left panel).

In addition to the soluble IL-7R, we measured IL-7 protein values in plasma from adults and children after aHSCT. The median IL-7 in plasma from adults was 7.36 pg/mL at 1 month versus 6.50 pg/mL at 12 months after aHSCT. In plasma from children, the corresponding values were 6.85 pg/mL and 31.05 pg/mL, respectively (Figure 3). The median IL-7 plasma level for controls (adults) was 4.70 pg/ml and 5.81 pg/mL (for children; Additional file 1: Figure S1, left panel). Regression analysis showed no significant correlation between sIL-7R and IL-7 values in plasma from patients (Additional file 1: Figure S1, right panel).

### Effect of donor age and conditioning on sIL-7R and IL-7 levels

Adults with stem cell donors above 30 years of age tend to have higher sIL-7R values at 2 months than to those receiving grafts from younger donors (p = 0.06). In contrast, adults with donors aged < 30 years had higher IL-7 levels at 6 months than those with older donors (p < 0.05, Additional file 2: Figure S2). Patients conditioned with RIC exhibited lower sIL-7R values 1 month after HSCT as compared to patients conditioned with MAC (p = 0.03, Additional file 2: Figure S2). There were no statistically significant differences between the patients who received RIC and MAC regarding IL-7 levels.

### Effect of donor, HLA-match, ATG, and stem cell source on sIL-7R and IL-7 levels

**Figure 4:**
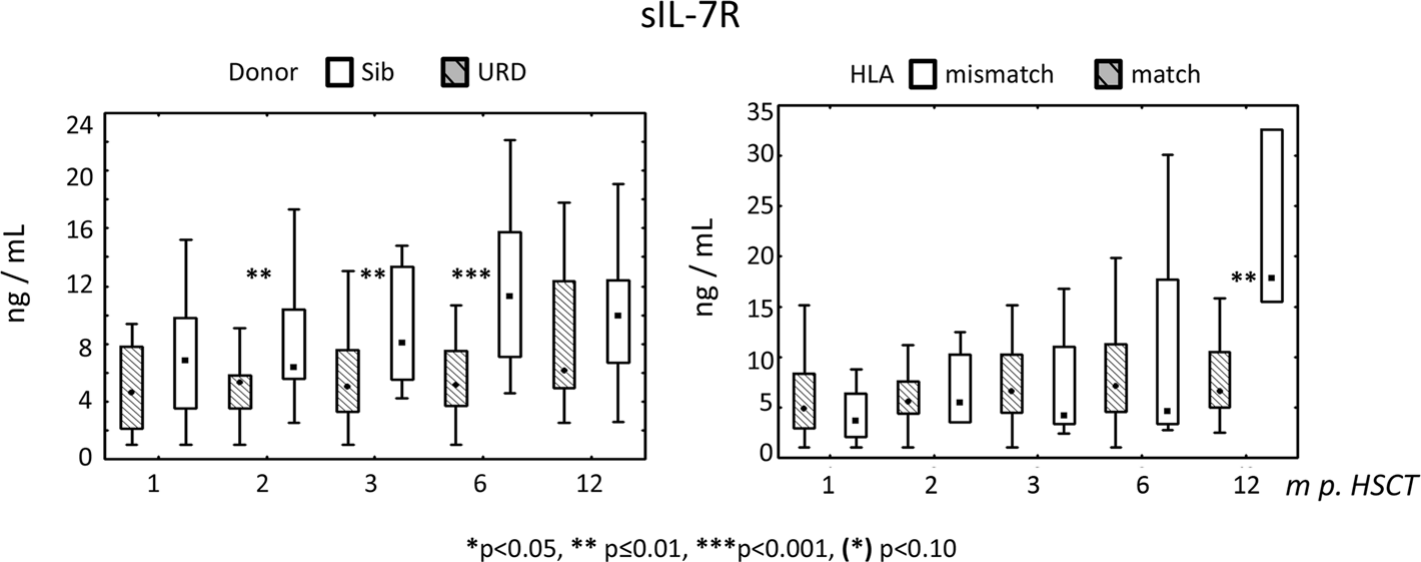
**sIL-7R levels after HSCT and analysis of clinical endpoints, i.e. type of donor (HLA-identical sibling (sib) or unrelated donor **[32]** and HLA-match versus HLA-mismatch.**

sIL-7R values were higher at 2, 3 (p < 0.01) and 6 months (p < 0.001) if the transplant was from an HLA-identical sibling rather than a MUD (Figure 4). Plasma from recipients of HLA-mismatched grafts exhibited elevated sIL-7R value at 12 months as compared plasma from recipients of grafts from HLA-matched donors (p < 0.001) (Figure 4). sIL-7R values were significantly higher at 2 and 6 months after HSCT if the conditioning regimen did not contain ATG (p < 0.05) (Additional file 3: Figure S3).

In plasma from children, sIL-7R levels were associated with the stem cell source. Plasma from children who received bone marrow grafts rather than peripheral blood stem cell grafts showed increased sIL-7R levels at 3 months after HSCT (p = 0.04) (Additional file 3: Figure S3). Patients treated with donor lymphocyte infusion (DLI) had significantly higher sIL-7R levels 1 month after HSCT than those who were not treated with DLI (Additional file 3: Figure S3).

### Association of sIL-7R and IL-7 with post-HSCT complications

**Figure 5:**
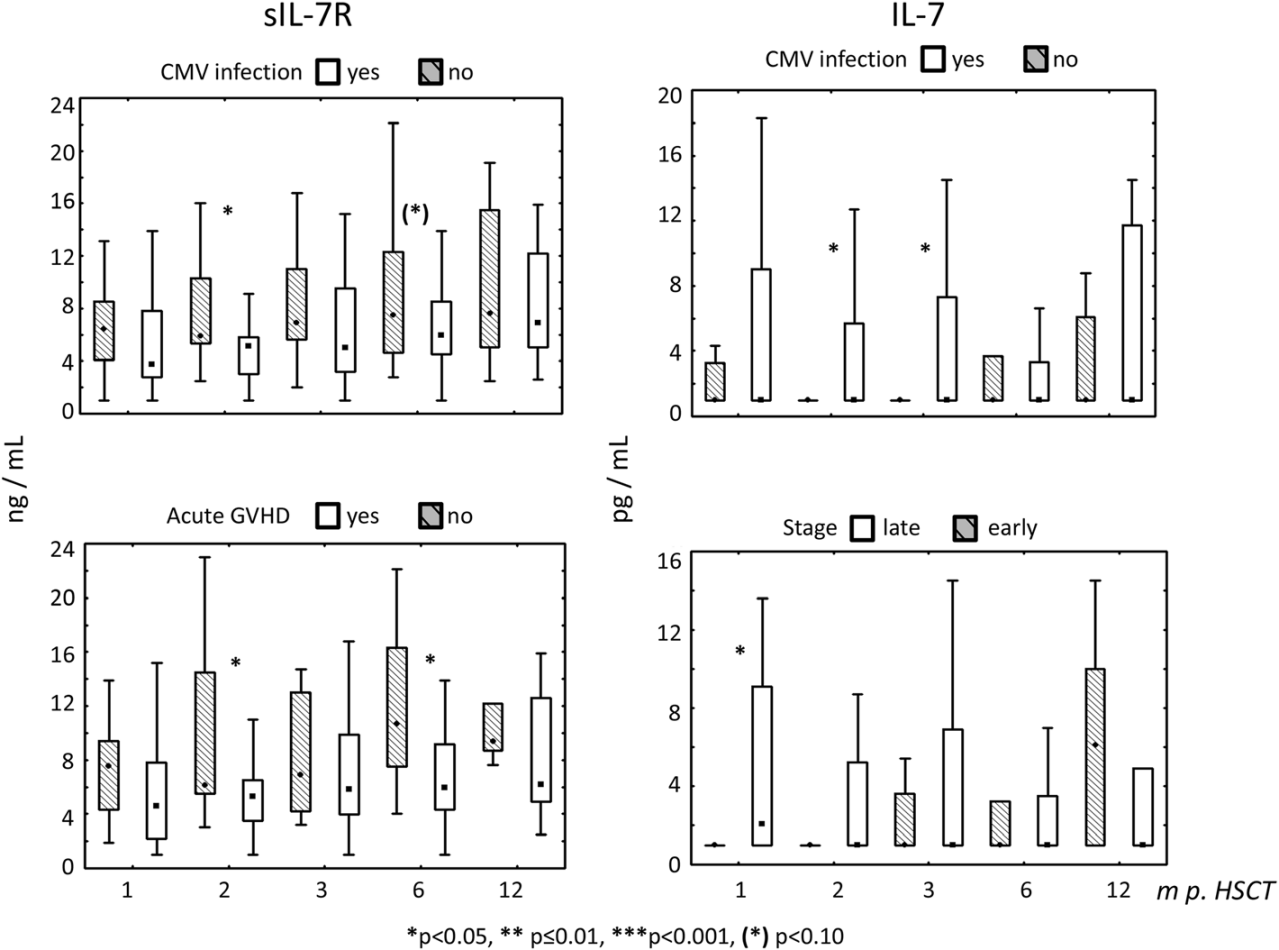
Levels of sIL-7R and IL-7 after HSCT, cytomegalovirus (CMV) reactivation (CMV PCR positivity) versus no CMV infection/reactivation, acute graft-versus-host disease (GVHD) grades I–IV versus no GVHD and stage of disease: early versus late.

We determined whether IL-7 or sIL-7R levels were associated with clinically and biologically relevant events in the course of HSCT in adults and children. Patients with acute GVHD of grades I–IV had lower levels of sIL-7R at 2 and 6 months after HSCT than those without any acute GVHD (Figure 5).

Plasma from patients with CMV infection exhibited lower sIL-7R values at 2 months after HSCT as compared to patients without CMV infection (p < 0.05). Plasma from patients with CMV infection showed higher IL-7 protein values at 2 and 3 months after HSCT than those without CMV infection (p = 0.02) (Figure 5). Plasma from patients with late-stage disease had higher IL-7 levels in the first month after transplantation as compared to patients with early disease (p < 0.05) (Figure 5).

### Multivariate analysis

In multivariate analysis of factors affecting the levels of sIL-7R at one month after HSCT, we found that acute GVHD was the only factor that affected sIL-7R levels (Table 2). At 3 months after HSCT, there was a correlation between the absence of acute GVHD and the use of a sibling donor and higher levels of sIL-7R. At 3 and 6 months after transplantation, use of stem cells from sibling donors was the factor that was most strongly correlated to higher levels of sIL-7R. At 12 months after HSCT, there was a correlation between receipt of an HLA-mismatched graft and high levels of sIL-7R.

**Table 2:** Multivariate analysis of factors affecting levels of soluble IL-7 receptors (sIL7R) at different time points after HSCT

**Factor**	**RH**	**95% CI**	**p-value**
**1 month after HSCT**			
**Sibling donor**	1.23	0.95–1.61	0.13
**aGVHD**	0.76	0.58–0.99	< 0.05
**2 months after HSCT**			
**Sibling donor**	1.31	1.01–1.70	< 0.05
**CMV infection**	0.83	0.64–1.08	0.17
**aGVHD**	0.70	0.55–0.90	< 0.01
**3 months after HSCT**			
**Sibling donor**	2.23	1.34–3.71	0.004
**aGVHD**	0.91	0.70–1.20	0.50
**ATG**	1.66	0.99–2.78	0.06
**6 months after HSCT**			
**Sibling donor**	1.86	1.21–2.86	< 0.01
**aGVHD**	0.77	0.58–1.04	0.09
**ATG**	1.30	0.83–2.04	0.26
**12 months after HSCT**			
**Sibling donor**	1.30	0.97–1.74	0.09
**HLA-Mismatch**	2.12	1.59–2.82	< 0.001

*Abbreviations*: *RH* relative hazard, *CI* confidence interval, *aGVHD* acute graft-versus-host disease, *ATG* anti-thymocyte globulin, *CMV* cytomegalovirus.

In multivariate analysis of factors affecting IL-7 levels, we found that late disease (beyond CR1) was associated with higher levels of IL-7 and that the use of bone marrow as the stem cell source was associated with lower levels of IL-7 at 1 month after HSCT. At 6 months after transplantation, the use of stem cells from a sibling donor was associated with higher levels of IL7 (Table 3).

**Table 3:** Multivariate analysis of factors affecting levels of soluble IL-7 at different time points after HSCT

**Factor**	**RH**	**95% CI**	**p-value**
**1 month after HSCT**			
**Late disease**	1.32	0.99–1.76	0.07
**BM**	0.73	0.55–0.98	0.04
**6 months after HSCT**			
**Sibling donor**	1.38	1.02–1.86	< 0.05

*Abbreviations*: *RH* relative hazard, *CI* confidence interval, *BM* bone marrow.

## Discussion

The motivation for examining soluble IL-7R after HSCT was threefold. First, increased sIL-7R levels have been shown to be associated with an increased risk of developing autoimmune responses [18] and we hypothesized that altered levels of sIL7R may drive GVHD. Secondly, increased soluble IL-7R has been shown to bind free IL-7 and to inhibit IL-7 signaling (and therefore immune effector functions) in CD8^+^ T-cells from patients with infections [33]; inhibition of IL-7, via binding to soluble IL-7R, could potentially impact on GVHD development. Thirdly, increased sIL-7R has been associated with improved immune reconstitution and immune competence in patients with HIV infection [34]. All three biological scenarios, i.e. immune reconstitution, resistance to infection, and higher risk of developing autoimmune responses are clinically relevant after HSCT. Up to now, it has not yet been well defined which cell type or tissue is responsible for generating sIL-7R. The soluble IL-7R could be generated by shedding from cells or by splicing of the IL-7r associated with a polymorphism in the IL-7R gene (rs6897932) [18-20].

Recipients of grafts from HLA-identical siblings showed higher sIL-7R levels than recipients of grafts from URD (unrelated donors) (Figure 4). This difference was not only significant in the univariate analysis but also in the multivariate analysis at 2, 3, and 6 months and with a trend at 12 months after HSCT (Table 2). It is possible that minor histocompatibility antigens (mHags) contributed to immune reconstitution and increased sIL-7R levels after HSCT. Poor immune reconstitution, including low sIL-7R plasma levels, may be associated with increased risks for infection in recipients of grafts from URD as compared to recipients of HLA-identical sibling grafts [35].

Earlier studies showed a close correlation between CMV infection and GVHD [36,37]. A significant finding in the univariate and the multivariate analysis in the current study was the decreased level of plasma sIL-7R in patients with any grade of acute GVHD (Figure 5, Table 2). Acute GVHD and also chronic GVHD have a profound effect on immune functions after HSCT [38,39] including increased risk of CMV infection: In the univariate analysis, patients with CMV infection exhibited lower levels of sIL-7R at 2 months after HSCT as compared to patients without CMV infection. This is the timeframe when most patients experience CMV reactivation after HSCT. Immune responses to herpesviruses in general, and CMV infection in particular may trigger acute GVHD. Not mutually exclusive, GVHD, may also pave the way for CMV infection, which delays immune recovery and increases risk of infections [40,41] after HSCT. sIL-7R and CMV infection was not significant in the multivariate analysis, which may suggest that GVHD is more important than CMV infection leading to decreased sIL-7R plasma levels.

We also found a tendency of lower sIL-7R levels in plasma from patients treated with ATG (Table 2). We treat all recipients of unrelated bone marrow grafts with ATG to prevent GVHD [42] at our center. In addition, patients with non-malignant disorders are also treated with ATG, since they do not benefit from GVHD. ATG has a prolonged effect on T-cell immune reconstitution and may therefore interfere with the generation of (soluble) IL-7R. The use of ATG also affects the rate of infections after HSCT in a dose-dependent fashion [28]. The data from the present study suggest that circulating T-cells (the numbers of which are reduced upon ATG treatment) contribute substantially to generation of soluble IL-7R generation, particularly since IL-7R expression is associated with T-cell maturation and differentiation [10].

Reduced levels of IL-7 protein were also identified in plasma from recipients of bone marrow as compared to patients who received peripheral blood stem cell transplants (Table 3). These two transplant types have different composite of the graft, which may be biologically relevant for IL-7 and soluble IL-7R production as well as IL-7 consumption. Blood cell grafts contain ten times more T-cells and NK-cells than bone marrow grafts [43,44], supporting the notion that sIL-7R is produced from circulating immune cells. Decreased sIL-7R levels were also identified in plasma from recipients of grafts from unrelated donors as compared to recipients of grafts from HLA-identical siblings. Furthermore, there was a correlation between acute graft-versus-host disease—and to some extent also CMV infection after HSCT—and lower sIL-7R levels.

Three independent studies showed that elevated sIL-7R is associated with an increased risk of to develop autoimmunity, a situation which maybe at first glance counter-intuitive: since soluble IL-7R may bind free IL-7 and neutralizes its effects [16]. Two alternative mechanism, not mutally exlusive, could explain the increased risk of developing autoimmunity due to elevated sIL-7R. The sIL-7R/IL-7 complex may serve as a buffer system and will first neutralize free IL-7. Serum IL-7 levels (which are one tenth of sIl-7R levels) are tightly controlled [45]. Firstly, IL-7, complexed to the sIL-7R, could be released later from its (soluble) receptor and drive autoimmune responses. Secondly, sIL-7R/IL-7 complexes may be more potent in driving expansion of CD8^+^ T-cell subsets (in murine experiments) [46]. It could very well be that IL-7, complexed to sIL-7R, delivers a more potent signal to the cell-bound IL-7R, an event which would be even more accentuated in ‘hypersensitive’ autoreactive T-cells [33]. To summarize, the IL-7/sIL-7R complex represents a double-edged sword: free IL-7 supports immune reconstitution and promotes increased immune reactivity in infections [47,48]; yet free IL-7 may also drive GHVD [3]. ‘Neutralized’ IL-7, by binding to the sIL-7R may not accessible to IL-7R-positive immune cells; this situation may be associated with increased risk of infections. Subsequently, IL-7, released from the sIL-7R, may be available to antigen-specific T-cells and ensure T-cell survival, which may also include immune cells mediating GVHD. Until now, the detailed dynamic of sIL-7R and IL-7 interaction in *ex vivo* collected clinical material has not been determined, yet a biologically and clinically relevant time frame to test for sIL-7R would be monthly within the first three month after HSCT; a time frame with a high risk to develop CMV infection and/or GVHD.

## Conclusions

Our data suggest that lower sIL-7R may be associated with increased risk of GVHD, i.e. that sIL-7R is not available in suffient amounts to serve as the IL-7 ‘buffer system’. Measurement of sIL-7R plasma levels, in combination with IL-7, may aid to identify individuals at higher risk to GVHD and potentially CMV infection.

## Competing interests

The authors declare that there have no competing interests.

## Authors’ contributions

TP carried out analyses and performed statistics, processed patient samples and wrote the manuscript, LR carried out analyses and established the sIL-7R ELISA, MR performed statistical analyses, BO was involved in patient recruitment, clinical management and data interpretation; NKV was involved in sample procurement and analyses, RA performed flow cytometric analyses, IE was involved in study design, data analyses and writing the manuscript, JW and AGJ were responsible for children’s care management, sample procurement, data analyses and interpretation, IM was responsible for flow cytometry, OR was involved in patient care and management, data analysis, study design and writing the manuscript, MM was responsible for the study design, data analyis and wrote the final version of the manuscript. All authors read and approved the final manuscript.

## Supplementary Material

Additional file 1: Figure S1 Plasma IL-7 and soluble CD127 levels in healthy children (top) and adults (bottom). B No correlation between plasma IL-7 and IL-7R levels in children (top) and adults (bottom) after aHSCT. R^2^, goodness of fit. Click here for file

Additional file 2: Figure S2 Analysis of clinical endpoints and IL-7/soluble IL-7R after HSCT: increased IL-7 is associated with younger donor (<30y, 6months) and RIC (12 months after HSCT), there is a trend for increased sIL-7R with the age of the donor (>30y) and MAC. * = p < 0.05. Click here for file

Additional file 3: Figure S3 Plasma sIL-7R levels after aHSCT and analysis of clinical endpoints, effect of DLI (donor lymphocyte infusion) compared to no DLI; effects of sources: PBSC (peripheral blood stem cell) vs. BM (bone marrow) vs. CB (cord blood) and immunosuppression with ATG (antithymocyte globulin) or not. * = p < 0.05, ** = p < 0.01. Click here for file
