# Supplementary figures and images for: Reduced plasma levels of soluble interleukin-7 receptor during graft-versus-host disease (GVHD) in children and adults
Source: BMC Immunol. 2014 Jun 19;15:25. doi: 10.1186/1471-2172-15-25 (PMC4074150; doi:10.1186/1471-2172-15-25)

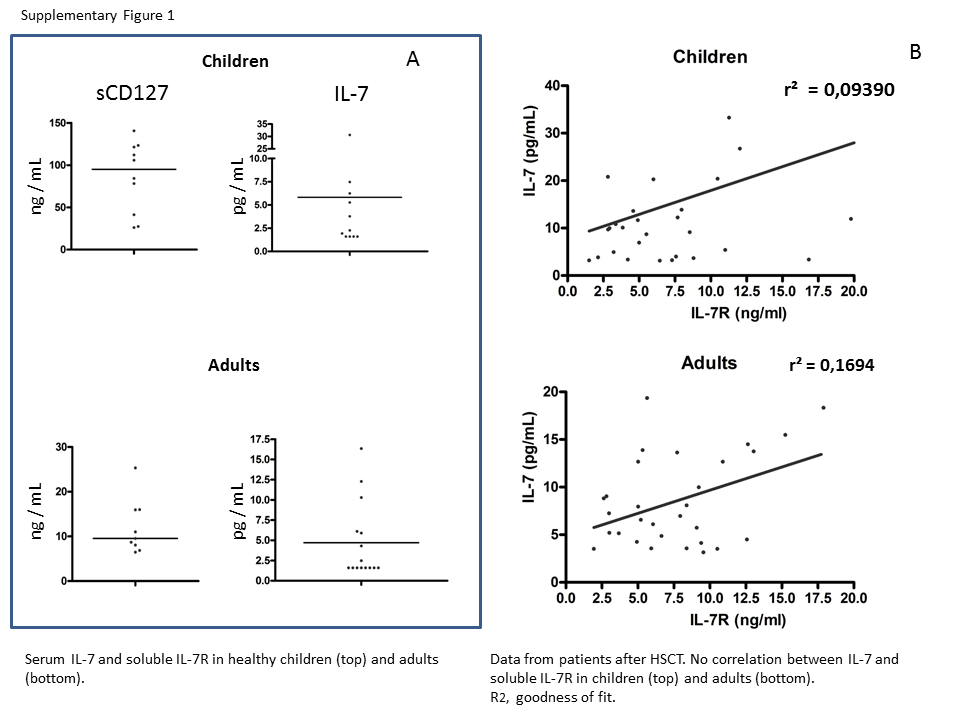

Supplement: Additional file 1: Figure S1 — Plasma IL-7 and soluble CD127 levels in healthy children (top) and adults (bottom). B No correlation between plasma IL-7 and IL-7R levels in children (top) and adults (bottom) after aHSCT. R2, goodness of fit. [file 1471-2172-15-25-S1.tiff]

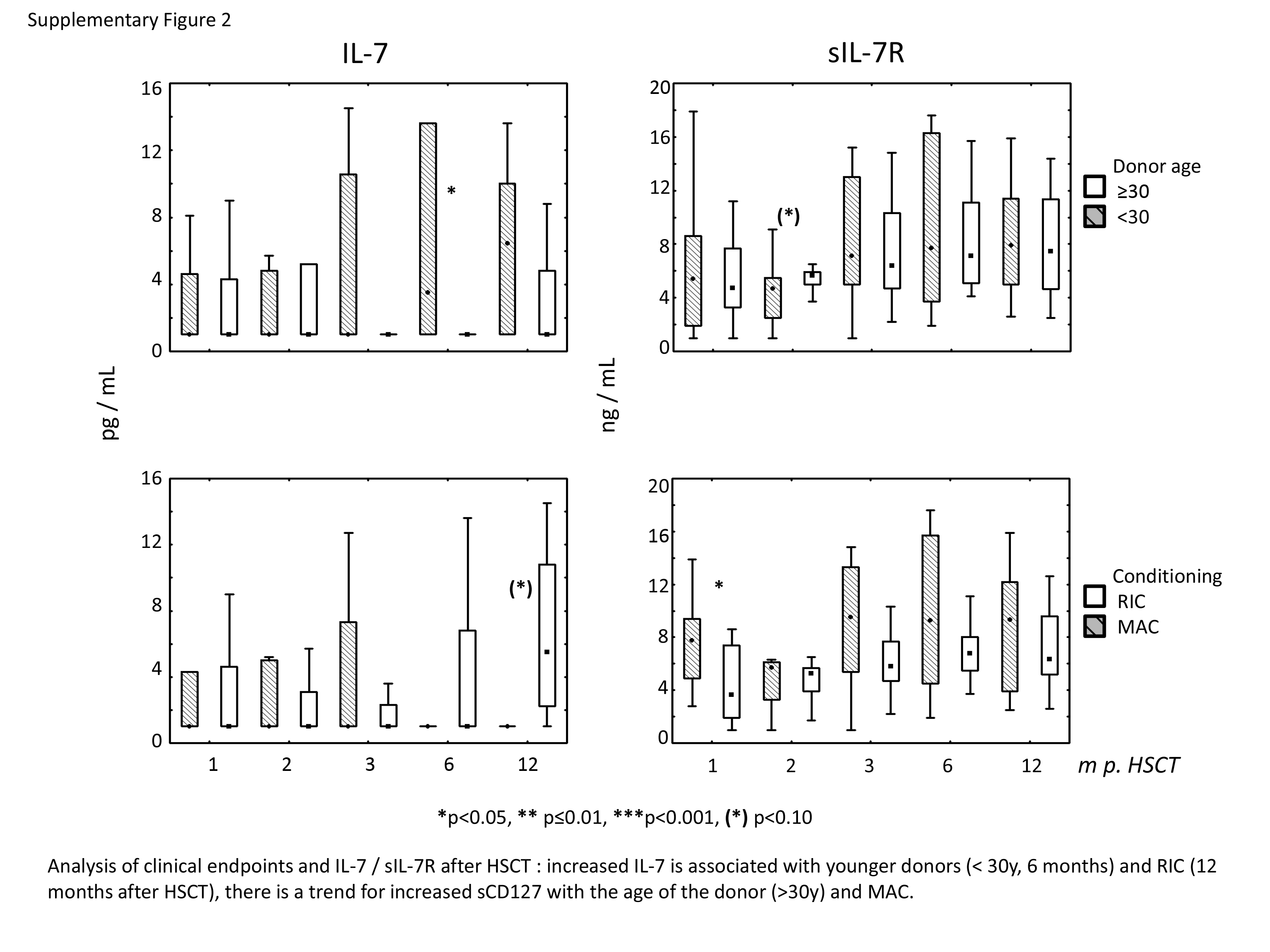

Supplement: Additional file 2: Figure S2 — Analysis of clinical endpoints and IL-7/soluble IL-7R after HSCT: increased IL-7 is associated with younger donor (<30y, 6months) and RIC (12 months after HSCT), there is a trend for increased sIL-7R with the age of the donor (>30y) and MAC. * = p < 0.05. [file 1471-2172-15-25-S2.tiff]

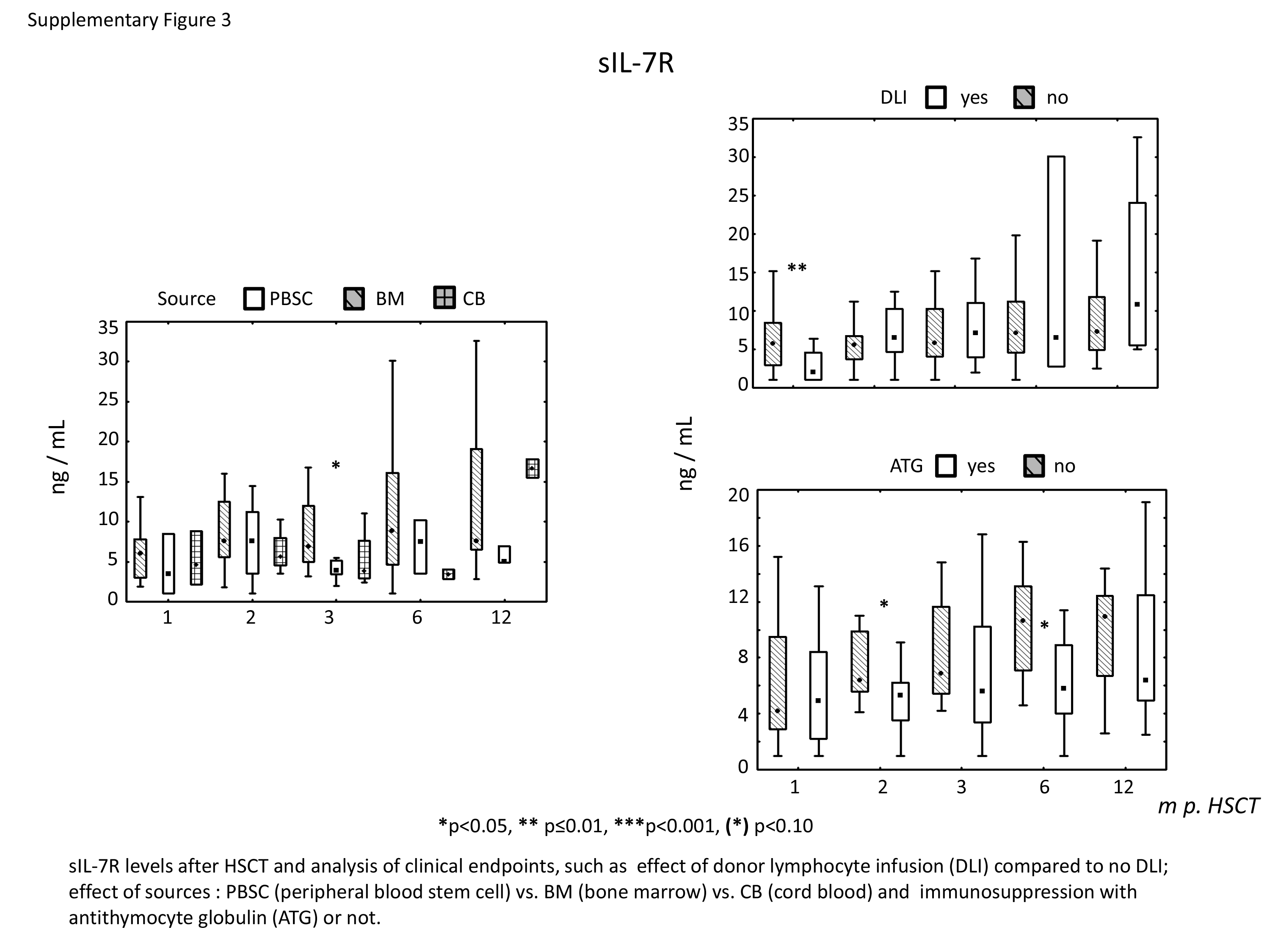

Supplement: Additional file 3: Figure S3 — Plasma sIL-7R levels after aHSCT and analysis of clinical endpoints, effect of DLI (donor lymphocyte infusion) compared to no DLI; effects of sources: PBSC (peripheral blood stem cell) vs. BM (bone marrow) vs. CB (cord blood) and immunosuppression with ATG (antithymocyte globulin) or not. * = p < 0.05, ** = p < 0.01. [file 1471-2172-15-25-S3.tiff]
